# MASSAI: Multi-agent system for simulating sustainable agricultural intensification of smallholder farms in Africa

**DOI:** 10.1016/j.mex.2023.102467

**Published:** 2023-10-30

**Authors:** Powell Mponela, Bao Le, Sieglinde Snapp, Grace B. Villamor, Lulseged Tamene, Christian Borgemeister

**Affiliations:** a Center for Development Research, University of Bonn, Genscherallee 3, Bonn 53113, Germany; b CGIAR Research Program on Grain Legumes and Dry Cereals (CRP GLDC), International Center for Agricultural Research in the Dry Areas (ICARDA), 2 Port Said, Victoria Sq., Ismail El-Shaaer Building, Maadi, Cairo, Egypt; c Department of Plant, Soil and Microbial Sciences, Michigan State University, 1066 Bogue St., East Lansing, MI 48824, USA; d New Zealand Forest Research Institute, Ltd (Scion), Te Papa Tipu Innovation Park, 49 Sala Street, Rotorua, 3046, New Zealand; e International Center for Tropical Agriculture (CIAT), Gurd Sholla Area, Bole Sub-city, Woreda 6, Addis ababa P.O. Box 5689, Ethiopia

**Keywords:** Re-orienting farm input subsidy, Farmer behaviour, Nutrient balance, Farm productivity, MASSAI: Multi-Agent System for Simulating Agricultural Intensification

## Abstract

The research and development needed to achieve sustainability of African smallholder agricultural and natural systems has led to a wide array of theoretical frameworks for conceptualising socioecological processes and functions. However, there are few analytical tools for spatio-temporal empirical approaches to implement use cases, which is a prerequisite to understand the performance of smallholder farms in the real world. This study builds a multi-agent system (MAS) to operationalise the Sustainable Agricultural Intensification (SAI) theoretical framework (MASSAI). This is an essential tool for spatio-temporal simulation of farm productivity to evaluate sustainability trends into the future at fine scale of a managed plot. MASSAI evaluates dynamic nutrient transfer using smallholder nutrient monitoring functions which have been calibrated with parameters from Malawi and the region. It integrates two modules: the Environmental (EM) and Behavioural (BM) ones.

• The EM assess dynamic natural nutrient inputs (sedimentation and atmospheric deposition) and outputs (leaching, erosion and gaseous loses) as a product of bioclimatic factors and land use activities.
• An integrated BM assess the impact of farmer decisions which influence farm-level inputs (fertilizer, manure, biological N fixation) and outputs (crop yields and associated grain).
• A use case of input subsidies, common in Africa, markedly influence fertilizer access and the impact of different policy scenarios on decision-making, crop productivity, and nutrient balance are simulated. This is of use for empirical analysis smallholder's sustainability trajectories given the pro-poor development policy support.

## Graphical abstract

**Figure:**
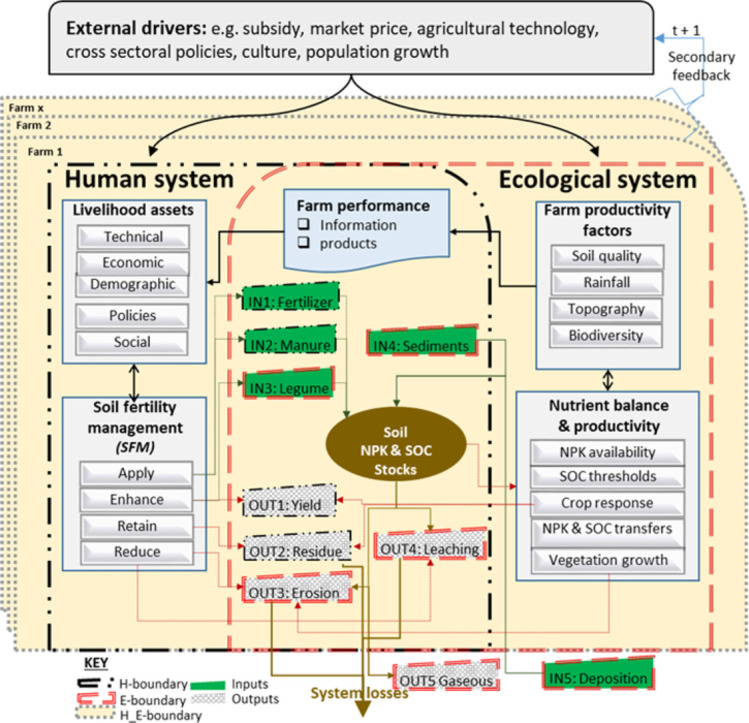

Specification table

**Table:** 

Subject area:	Agricultural and Biological Sciences
More specific subject area:	Soil fertility
Method name:	MASSAI: Multi-Agent System for Simulating Agricultural Intensification
Name and reference of original method:	FarmDESIGN [1]. Multi-objective optimization and design of farming systems. *Agricultural Systems*, **110**, 63–77, (At: http://linkinghub.elsevier.com/retrieve/pii/S0308521 × 12000558.) NUTMON: NUTrient MONitoring for Tropical Farming Systems [2]. A decision-support model for monitoring nutrient balances under agricultural land use (NUTMON). *Geoderma*, **60**, 235–256, (At: https://linkinghub.elsevier.com/retrieve/pii/001670619390029K).
Resource availability:	https://github.com/powellmponel/MASSAI

## Introduction

The concept of sustainable agricultural intensification (SAI) is traced back to the mainstreaming of sustainability in development programs in 1980s [3] and a decade later with specific focus on agriculture [4]. Research tools and policies that support SAI have been challenging to develop, despite the critical need to intensify production while maintaining the integrity of the resource base. Research and international agenda on transformation of ecosystems and their implications on livelihoods focus on large scale natural disasters, disturbances to large ecosystems and regional economic shocks [5]. Much as these single events erode long-term achievements, during the Anthropocene era, the day to day human actions lead to land degradation and put at risk the livelihoods of land dwellers especially of smallholder farmers [6]. In the past two decades, several conceptual and theoretical frameworks and programs have been developed to conceptualise the sustainability of agricultural systems under smallholder farming conditions [[7], [8], [9], [10]]. Still, there are discussions on how to analyse dynamic reverse causality among human and ecological sub-components in smallholder farming systems, given the multitude of inputs and outputs, and their interactions and feedbacks [11].

Achieving SAI is a broad goal aimed at ensuring efficient use of resources for improved productivity, with equal attention to equity and environmental services [12]. Striking the balance is still considered as one of the major research and development challenges since it requires an understanding of how ecosystems function under changing environment and socio-economic constraints [13]. As Schreinemachers and Berger [14] found, sometimes a higher productivity in terms of yield is not the main goal for small-scale farmers. This is the case because smallholders are overly constrained by multiple factors including labour, capital, fertilizers, pesticides and herbicides and sometimes by insecurity over land rights that they have to overcome. Researching options for addressing these constraints is methodologically challenging with regards to (1) the complex human-environment interactions among factors, (2) uncertainties caused by that complexity, (3) the long-term perspective of sustainability research, and (4) externalities and trade-offs over space, time and social groups [15].

Of the five SAI components including breeding, nutrient input and recycling, pest and disease management, labour and mechanisation, and markets, it is purported that nutrient and fertility decline is the primary biophysical limitation to sustainability attainment [[16], [17]]. In smallholder cropping systems of East and southern Africa, without proper management interventions, continuous cultivation lead to depletion of major nutrients nitrogen (N), phosphorus (P) and potassium (K) as well as soil organic carbon (SOC), rendering farming systems less productive [18].

In the absence of established long-term trials, detailed typical farm analysis (TFAs) of nutrient balances for a few archetypical (cluster centroid) farms have been widely conducted to analyse (non-) performance using bio-economic models such as NUTMON and FarmDESIGN [[1], [2]]. These TFA and their survey tools are empirically parameterised to capture nutrient flows but are limited to the use of static entities of agricultural enterprise and land units. Without the decision-making component and spatio-temporal considerations, they fall short to capture dynamic feedback, evolution of processes and system states, that are essential to understand social-ecological sustainability [15]. In addition, by using a few archetypical farms on the assumption of representativeness, they fail to allow for and capture autonomous actions by heterogeneous farmers that interact within landscapes and communities.

Therefore, this study uses empirical sub-models to addresses gaps in bio-economic research on decision with geolocated and integrated datasets from plot level, soil, agronomic inputs and practices to policy drivers. In addition to plot level, a comprehensive review of scientific work in southern Africa and used to update the ecological modules for nutrient balance estimation. By integrating behavioural and ecological modules, we build the multi-agent system (MAS) which is well-suited for analysing social-ecological system's (SES) sustainable development [15] and is used for comprehensive *ex ante* assessment of impacts of policy interventions on land use and cover [[19], [20], [21], [22], [23]]. We analyse and simulate the sustainability impacts of farm input subsidy program in terms of influence on human behaviour, farm productivity and nutrient balances in maize mixed farming system of Malawi.

## Methodology

### Framework, empirical data and policy scenarios

#### MASSAI analytical framework

The study applies the overview, design concepts and details + decision (ODD + D) MAS protocol by Railsback and Grimm [24]. The ODD+D's study intent, characteristics of the study units and processes and the procedure are summarised in Table 1 and detailed in table S1. Structurally and functionally, the maize mixed smallholder farming system of East and southern Africa is characterised by an environment, objects and agents, relations between all the entities, a set of operations that can be performed by the entities, and the changes of the agro-ecosystem in time due to these actions [25].

**Table 1:** Input and output flows for major plant nutrients (NPK: nitrogen, phosphorus, potassium) and carbon (C) for Ecological and Behavioural Modules (EM & BM).

Pathways	Agent action and linked sub-module(s)	Data*	Models*
IN1_-NP_	_BM-_Farmer decides to apply inorganic fertilizer	Survey	
IN2_-NPK&C_	_BM-_Farmer decides to apply manure and retain residues	Survey Review Soil maps	
	_EM_-Decomposition of manure and residues (including roots) and soil organic matter		[26]
IN3_-N_	_BM-_Farmer decides to plant legumes	Survey Review	
	_EM-_Biological nitrogen fixation		
IN4_-NPK&C_	_EM-_Sediment delivery ratio during OUT3_-erosion_	Review Survey	[27]
	_BM-_Farmer use soil and water conservation (SWC) measures to retain eroded soil		
IN5_-NPK_	_EM-_Wet and dry atmospheric deposition from rain & wind	Review	[28]
OUT1_-NPK_	_BM-_Farmer enhances crop growth and yield	Survey	
	_EM-_Estimated through crop-yield sub-module		
OUT2_-NPK_	_BM-_Farmer incorporates or take out crop residues	Survey	
	_EM-_Estimated from crop-yield sub-module		
OUT3_-NPK&C_	_EM-_Soil EROSION sub-module	Review Soil maps	
	_BM-_Farmer uses SWC measures to reduce erosion		
OUT4_-NK_	_EM-_leaching sub-module	Soil maps Rain Survey	[29]
OUT5_-N&C_	_EM-_N-Denitrification and volatilisation.	Soil maps Rain Review	[29]
	_EM-_C-Estimated from SOM degradation		

⁎ Updated the submodules for NUTMON and FarmDESIGN; Reviews of other updated submodules and parameters are reported in Supplementary S3.

**Fig. 1:**
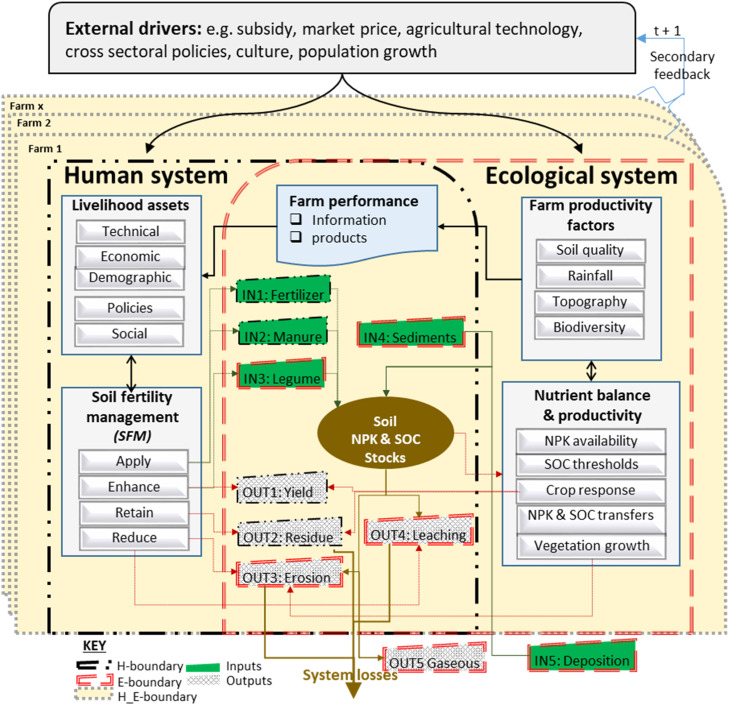
The Multi-Agent System for simulating Sustainable Agricultural Intensification (MASSAI) framework.

Both the human and ecological farm entities are inherently variable (Fig. 1). The households differ in demographics, resource endowments and nutrient input use, and are categorized into low organic and inorganic input (*farmtype1*), medium input (*farmtype2*) and high input (*farmtype3*) farms. In the ecological system, the plot and landscape features also vary, which, coupled with household attributes, frame the differentiated decisions on soil nutrient distributions, yields, nutrient balances and economic benefits among human and ecological agents.

The linkage between human and ecological components arises from the autonomous decisions by farming households as *agents* to apply, retain, reduce and enhance nutrient input-output flows such as fertilizer, manure and erosion (Fig. 1). Under real farming conditions, household resources, the preceding soil fertility and farm productivity informs the subsequent decision on types of activities and the perceptions about benefits that an individual has on the farmland. To model spatially explicit evolutions of the maize-mixed farming system as a coupled human-environment system at a landscape level, the conditions and interactions among farming households and the services and conditions of the land patches ought to be empirically calibrated.

Nutrient balance results from integration of human induced flows and ecological flows have several sustainability implications. Of major concern is that low nutrient input leads to low food production and soil degradation. On the other hand, excessive application of major nutrients especially N and P can lead to nutrient draining into the environment causing pollution and low profitability. The total soil nutrient (*X_soil_*) after a growing calendar year (*t+1*) is expressed as:

$$f\left(X_{soil}^{t+1}\right)=f\left(X_{soil}^{t}+\frac{1}{\rho CD}\left(\sum X_{in}^{t}-\sum X_{out}^{t}\right)\right)$$

Where *X* includes the nutrients NPK and the SOC, whilst *ρ, C* and D are soil density, coarse fragments factor and profile depth, respectively. The depth is set at 10 cm considering that the ridge spacing is 60, 75 and 90 cm and corresponding heights are 20, 25 and 30 cm [30]. Annually, farmers re-make the ridges by hand hoeing and minimise drudgery by scraping to the minimum depth possible. Moreover, fertilizer and manure are placed at 5 cm depth on the ridge as it has been established that such supplements largely contribute to nutrient stocks for the upper 0-10 soil layer [31].

The work builds on the human environmental systems (HES) framework [32] that has been operationalised to simulate socio-ecological performance of farming and other managed natural [[20], [22], [23], [33]]. To capture, analyse and present both human and ecological processes, a Multi-Agent System (MAS) for simulating Sustainable Agricultural Intensification (MASSAI) has been developed. MASSAI integrates two modules: The primary core module is the Ecological Module (EM), which assesses farm sustainability by evaluating natural material stocks and flows in the *environment*. For this, the static nutrient transfer functions from NUTMON and FarmDESIGN were adapted and updated with parameters from the study region (Table 1 and Supplementary S3).

The environmental *agent*, which is the farmland, hosts dynamic natural nutrient inputs like sedimentation and atmospheric deposition, and outputs like leaching, erosion and gaseous loses, that occur dependent on bioclimatic drivers and land use activities. The core EM assesses farm sustainability by evaluating natural material stocks and flows for the 10 m spatial resolution pixels across the landscape. The _CROP-YIELD_ sub-module is estimated using generalized linear model (GLM) and analyses bounded maize and legume yields of the plot given the household livelihood profile, land productivity, soil fertility management (SFM) technologies and policies.

The secondary module is the Behavioural Module (BM), which assesses nutrient flows arising when farmers as the active agents manage their farms to maximise productivity, through nutrient inputs: fertilizer, organic manure and legumes and outputs: yield and residue, which is contingent on available resources, farm conditions and policy interventions. For each input strategy, in a cropping season which is a calendar year in Malawi with uni-modal rainfall, farmers face two hurdles: first, whether to allocate available SFM to a plot/crop or not. Secondly, they decide to increase, maintain or reduce the amounts of inputs/outputs and land area to be covered. A double hurdle model is, thus, used, with the first using bi-logit model and the second using GLM. Both decisions are bounded by policies (input subsidy), natural capital (farm size, soil nutrient stocks, topographic position, land quality, crop land, livestock), farmer livelihood profile (labour, gender, age, education, communication, transport, income), social capital (group membership) and linkages among complementary SFM practices.

**Fig. 2:**
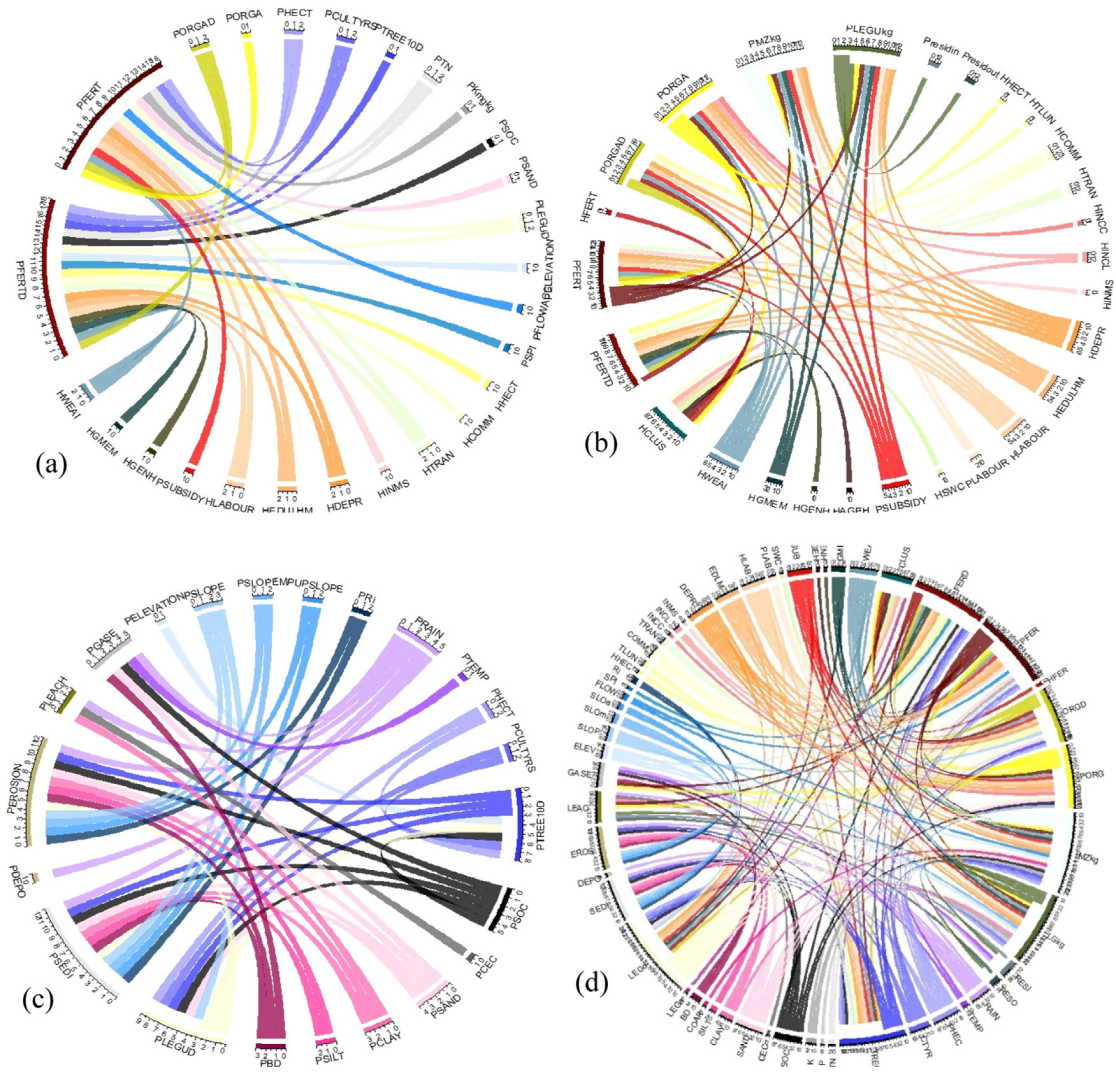
System integration: (a) direct influence fertilizer choice; (b) indirect among the human system components; (c) indirect among the ecological systems; and (d) through feedback loops among human and ecological systems. **NB:** The flow has the same colour as the driver; and there is a white space between flow and the target variable. The definitions of the variables are presented in supplementary Table s2. (e) without suffixes H_ for human and P_ for plot

EM and HM are integrated intersectionally, multi-stage, or feedback-loops (Fig. 2). Intersectionally in a sub-module (Fig. 2a) where several factors drive a process leading to the same outputs. Multi-stage within a core module (Fig. 2b and c), an output from one sub-module is an input in another such as IN3_-Legume_ influences IN1_-Fertilizer_. Feedback-loop integration (Fig. 2d), occurs as MASSAI updates farmer and farm attributes such as soil nutrient status, by subtracting outputs from inputs - giving the balance that is added to or subtracted from the existing nutrient stocks and affects subsequent flow processes. Integrated, there are system feedbacks and the external factors such as policies, climate change and markets that change the driving forces, resource availability and management capabilities and can accelerate, stabilise or reduce nutrient transfers, thereby influencing farm sustainability. Given that a representative sample of households and farmlands were used to train and validate the model, its application to the wider community was realised by generating the remaining populations and upscaled based on agent attributes and states using Monte Carlo approximation and GIS-based proportional up-scaling.

#### Sampling and data sources

A smallholder maize mixed farming system landscape composed of five villages in Ntcheu District, Malawi, that receive fertilizer and seed subsidy was chosen as a case use. The site, Nsipe, has been a research out-scaling area for agricultural intensification since 2013 (more details on the site on soil and landscape characterisation and usage of soil fertility management can be found in Mponela *et al*. [[34], [35]]). The study households, plots and sub-watersheds were drawn using multi-stage sampling. The household choice was dynamic as no prior attributes were available which allowed for a plausible understanding of households’ strategies [36]. Nested within the households, the plots belonging to those sampled were then enumerated for ecological studies. For integration of human and ecological sub-systems, a common geo-referenced sampling frame was used [37] for a sample of 250 households and their 451 plots (representing 17.1 % of the cultivated land).

Primary data was obtained through farm surveys of soil, biomass and crop yields, nutrient inputs and outputs, and households. Secondary data included spatial data of soil types [38], soil nutrient distributions [34], land cover and use [39], soil and vegetation reflectance derived from 10 m spatial resolution sentinel2 satellite imagery [40], and topography derived from 30 m Shuttle Radar Topography Mission (STRM) digital elevation model (DEM) [41]. A review was conducted to get contextualised parameters for updating empirical models (Supplementary Table S3).

#### Fertilizer subsidy impact scenarios and impact pathway

**Fig. 3:**
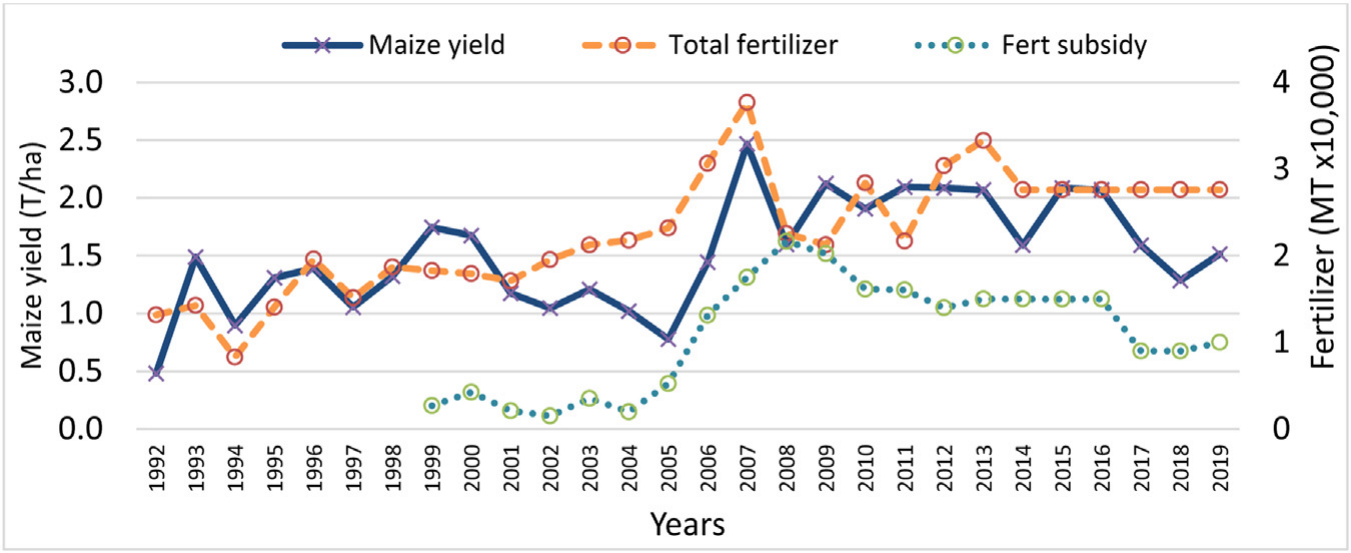
Trends of national average maize actual yields and fertilizer (Fert) subsidy between 1999 and 2019 in Malawi.

In determining the dynamic changes in usage of nutrient input technologies and the nutrient output processes, we examined the changes in exogenous policy actions but also controlled for the main internal factors. Among the various policies that most African governments provide, and communities appreciate as viable policy options to enable them to replenish lost nutrients and increase crop yields, the farm input subsidy program (FISP) is noticeably featured (Fig. 3). Efforts to improve productivity of smallholder farmers in Africa by replicating the lessons from Asia's green revolution proved futile with wide yield gaps and the associated endemic poverty traps [42]. In early 2000s, it was noted that gains from SAI technologies could not be realised mainly because the depletion of soil fertility was not addressed. In 2006, African governments agreed to increase public agricultural expenditure towards soil fertility improvement, which is still pivotal to the attainment of SDG targets [43]. In tandem with that, Malawi has been allocating substantial budgetary support to a farm input subsidy program since 2005, which resulted in a rise in fertilizer input and corresponding yield, but plateaued far below the optimal fertilizer requirement and potential maize yields for the past 15 years [[44], [45], [46], [47], [48]]. This makeCs Malawi a candidate for exploring strategies for repurposing subsidies as proposed by the World Bank [49].

Although the subsidy program is a pro-poor strategy aimed at reducing the nutrient gap, the sharing of coupon price and inputs makes it difficult to discern recipients from non-recipients, challenging an impact assessment. From our survey, we captured the total cost farmers paid for the fertilizer applied on a plot and divided with the market price to find the share of the subsidized input.

The share of subsidized fertilizer applied to maize plots for the 2016/2017 growing season averaged 28 %. This was used as a baseline, and all policy and static variables were held constant but progressively changing variables such as age of household head and the cultivation period for the plot were updated. By bringing other factors to the baseline levels and project their progressive natural changes, the effect of policy interventions is therefore additional and conditioned on existing conditions. Four scenario spectrums were constructed based on the projected trend incrementally increasing or decreasing over the 20-year simulation period as: (a) current subsidy contributing an average of 25 %, vs. political aspirations and alternative policies that aim at (b) reducing subsidy to around 15 %, (c) reducing to zero, and (d) providing universal subsidy which is the vision of the current government.

Fertilizer subsidy programs in Malawi have increased fertilizer access to many smallholders (Fig. 3), with associated feedbacks regarding market prices and, thus, access more broadly. The input subsidy affects the input prices with potential effects on output levels and the price bargaining power depending on the level of subsidy received by the farmer. Yet, farmers receiving subsidized “*free*” fertilizer might not be as committed to manage the crops as those who purchase the fertilizer, thereby having differentiated nutrient output flows.

Although there is no clear evidence on effect on subsidy on manuring, available evidence shows a high complementarity between fertilization and manure, with subsidy recipients apply comparatively less manure [50]. Hence, we hypothesized that those receiving subsidized fertilizers, by having an increased likelihood to access inorganic fertilizer at a cheaper price, would be less likely to invest in alternative nutrient sources such as manuring. However, since the subsidy scheme includes legume seeds, we hypothesized that farmers receiving subsidy would have a higher probability to plant legumes or allocate more land to legumes than non-recipients would.

### Programming MASSAI platform using NetLogo language

Using the ODD+D framework, the attributes of farmers and farms, their states, actions and processes and responses to changes in subsidy regimes were calibrated and implemented in the NetLogo MAS language [51]. We adopted the base code of the Land Use Dynamic Simulator (LUDAS) platform developed by Le [20] for payment of ecosystem services in agroforestry systems of Vietnam. Additional work was done to further customise LUDAS to the context of the study site within the maize mixed farming system and the overall policy and governance setting of the community. We built new modules (e.g., nutrient balance) and specify across all module's relevant variables, empirical parameterisation and verifications.

**Fig. 4:**
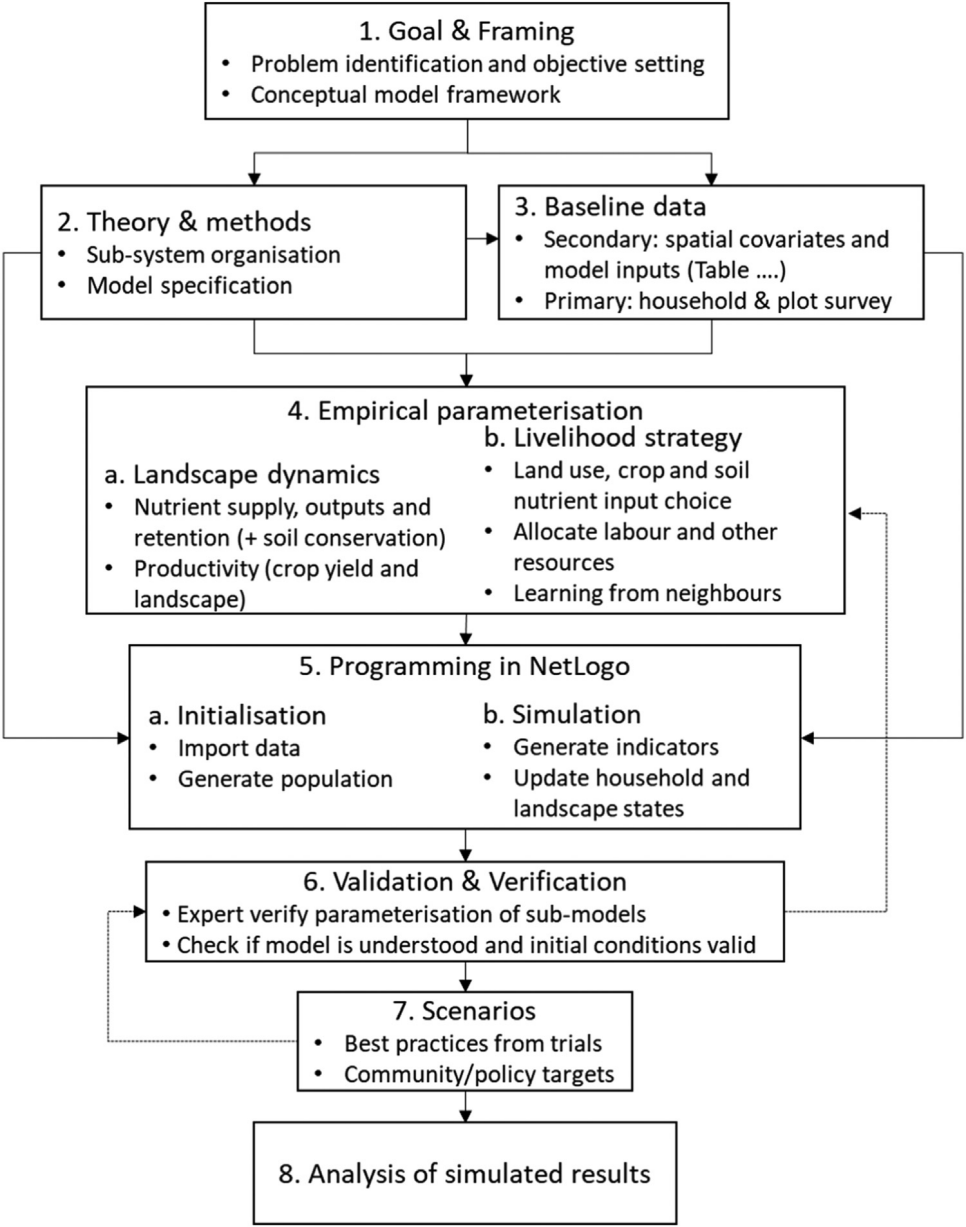
Workflow of MASSAI implementation process

The modelling steps and main procedures are presented in Fig. 4 and Box 1.

#### Initialisation

**Fig. 5:**
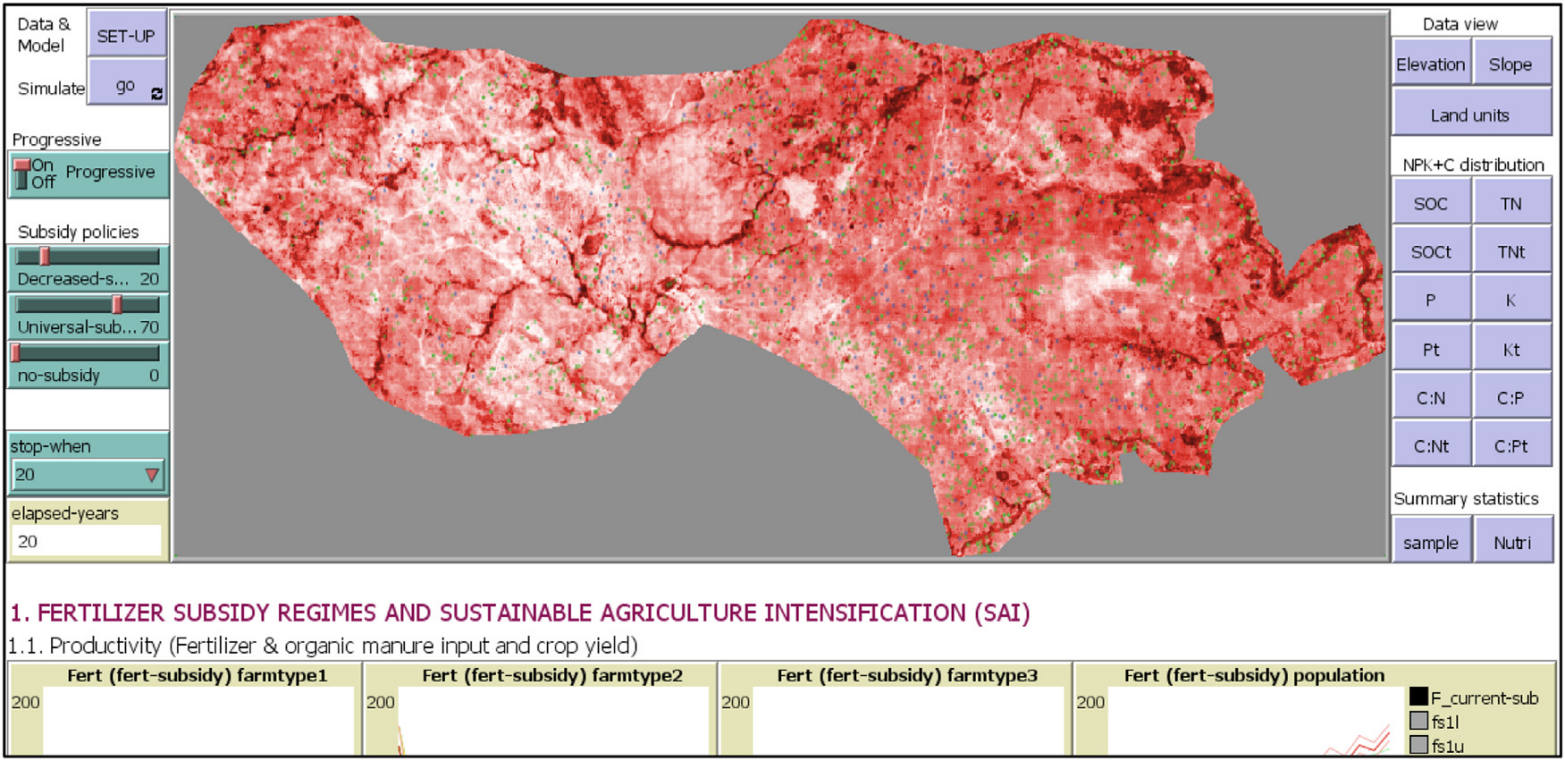
MASSAI interface in NetLogo

The first step in model development was initialisation (Fig. 4), when both spatial and household-plot data are loaded, the remaining population of households and their plots are generated in the unsampled landscape and the parameterisation of sub-modules were set-up forming a baseline scenario (Box 1). This is loaded using the ‘*SET-UP*’ button on the NetLogo user interface (Fig. 5). When designing the processes we recognise that although the ecological processes such as plant growth and decay are largely regulated by environmental factors, the states of factors are altered by human actions, resulting in trade-offs and synergies between ecological, economic and social sustainability measures [9]. Hence, the analytical platform is built on the hypothesis that the smallholder farmers as agents act autonomously but interact as they share the environment. Their combined actions when observed at community/landscape level emerge as fragmented patches with different management, productivity and underlying processes. Without spatial explicit attributes, the patches would be arranged forming a gradient that is usually averaged. Consequently, smallholder farming has been considered an unorganised complex system and a hybrid analytical approach that combines hypothesis driven experiments used for organised simple systems and statistical data driven observational studies used for complex unorganised systems is used [20].

Box 1 **Netlogo 6.2.0 main procedures for the MASSAI**

Alt-text: Unlabelled box

#### Simulation

The second step is the simulation (Fig. 4), operated by the ‘*go*’ button on the user interface (Fig. 5) that iteratively analyse the spatio-temporal changes in inputs and outputs, under different policy subsidy scenarios (Box 1). The simulated changes are based on the assumption that external and some internal factors that drive inputs and outputs progressively and dynamically change each year which is set ‘*on***’** using the switch ‘*Progressive***’**.

The interface features the sliders for adjusting the major nutrient input strategy, i.e., the fertilizer subsidy (Fig. 5). To explore effects of policy actions on adoption and intensification of SFM usage and the resulting impacts on productivity, nutrient balance and profitability, a causal relationship ought to be established. However, in cross-sectional studies real-life phenomena tend to have many confounders [52]. These are factors that correlate with both the dependent and independent variables, making it difficult to discern between association and effect [53]. In MAS models, the aim is to establish causal relationships, feedback loops, synergies and trade-offs among several factors; and confounders are inherent and a major threat to the validity of inferences [54]. There tend to be a triadic reciprocal causation, where resource endowments, behaviours and environmental states and processes all operate as interacting determinants that influence each other bidirectionally [55]. To avoid type I errors (false positives) of indicating a casual effect, several methodological approaches are used. In MAS, casual diagrams (Fig. 2), which are theoretical frameworks or impact pathways that provide a visual or theoretical model for distinguishing causation from association, are widely used [56]. Building on these, process-based models are specified with input and potential outcome model structure [54].

The stochasticity in the predicted input and output flows for the ABM were expressed using random bounded functions, with parameters allowed to vary randomly within the estimated confidence intervals than would be the case if the coefficient and margins-at-mean were used [[20], [54]]. For each scenario and replication, the stability was achieved by setting random seeds, thereby ensuring the same initialization and the same set of parameters. We run 10 replications which is the minimum number of recommended for complex ABMs with non-linear relationships between the input parameters and simulation output [57].

#### User interface and codes

**Fig. 6:**
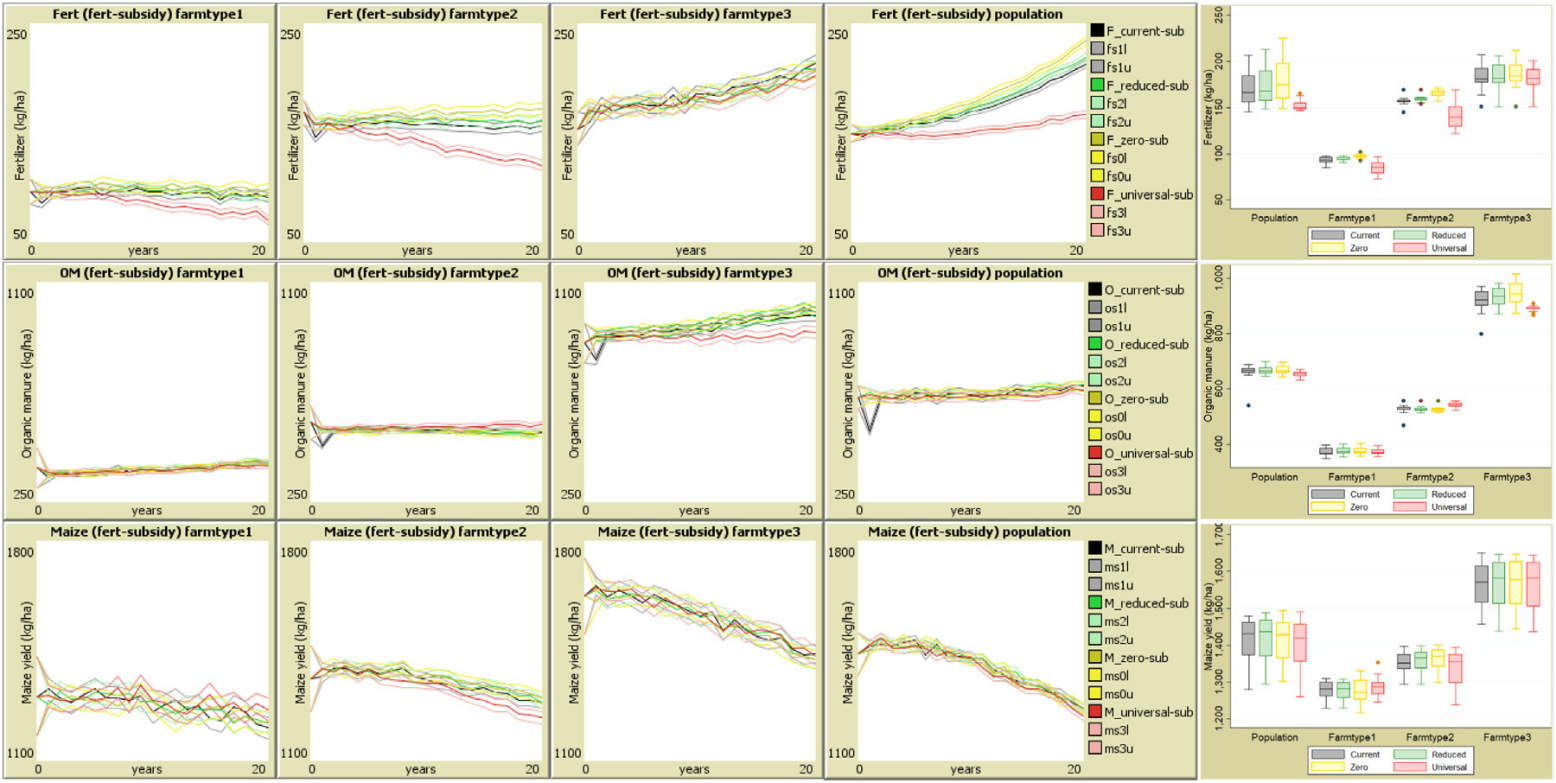
Yearly effects of subsidy regimes on fertilizer and manure input and the maize productivity. NB: input 1 (inorganic fertilizer- upper panel), input 2 (organic manure, OM – middle panel) and output 1 (maize yield – lower panel) for the entire population (second panel from right), and the farm types 1, 2 & 3. The darker middle lines are mean estimates for subsidy scenarios (_current-sub = current (28 %), _reduced-sub= reduced to 15 % as per trend, _zero-sub= reduced to zero, _universal-sub = universal increase to 70 %). The lighter lines are 5 % (_l) and 95 % (_u) confidence intervals. The appended box plots on the right shows the median and range of average predictions over the 20-year period.

**Fig. 7:**
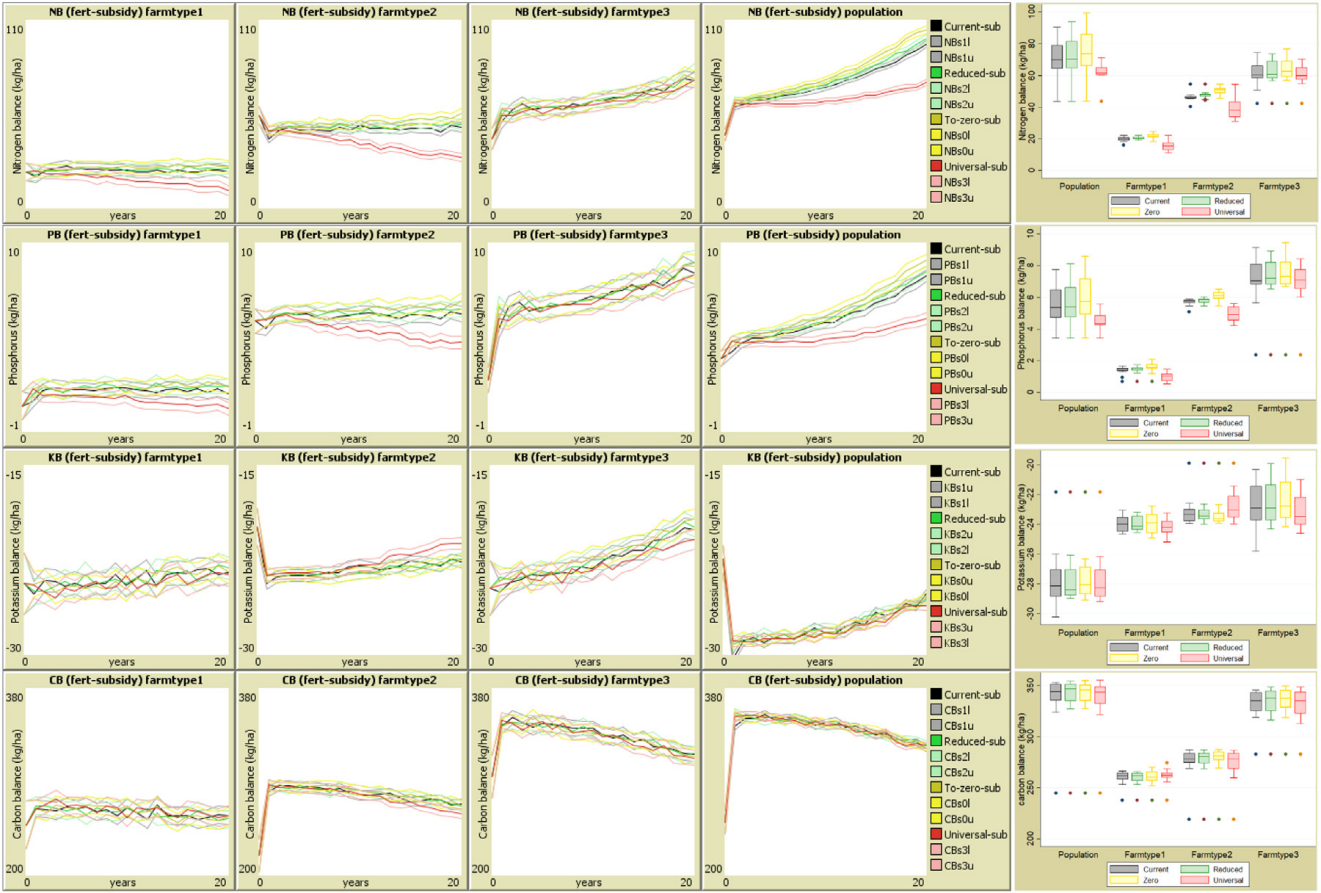
Interannual effects of subsidy regimes on partial nutrient balances. NB: Nitrogen- upper panel, phosphorus – second, Potassium – third and carbon – lower panel: for farm types 1, 2 & 3 and the entire population. The darker middle lines are mean estimates and the lighter lines are confidence intervals. The appended box plots on the right shows the median and range of average predictions over the 20-year period.Conclusion

The last step is the model output (Fig. 4). On the right of the interface, the user can view data and statistics after initialisation or at the end of simulation. The interface has a graphical results section which are used for trend reporting and exported for moving average analysis into a statistical software (Figs. 6 and 7). The iterative simulated outputs were averaged for each scenario, and the uncertainty was evaluated using confidence interval which is also indicative of the statistical differences between scenarios. After simulations, to compare the current policy with alternative regimes, we used the Bonferroni multiple comparison test which tests significance based on individual *p*-values between moving averages [58].

The main codes for MASSAI procedures are presented in Box 1. Detailed sub-procedures are saved separately in ‘*__includes*’ files and can be obtained on request from the corresponding author and downloaded from: https://github.com/powellmponel/MASSAI.

#### Model validation and sensitivity analysis

The sample dataset used for empirical estimations and predictions was obtained from 238 households with 451 plots, hence for the whole sample, the number of subjects per variable (SPV) is adequate, even if the rule of thumb of 10 SPV is followed. For instance, observation (451) to variable (21) ratio for maize yield estimation and prediction was 21:1 which gives reliable estimates. With yield model's SPV of 4, 8 and 6 for farmtypes 1, 2 and 3 respectively, the type-specific results may not reliably predict outside the dataset. However, for Monte Carlo simulations, recent studies have shown that the minimum SPV of two is adequate for estimation of regression coefficients, standard errors and confidence intervals [59]. Hence, for the type-specific analyses, the inclusion of a number of variables ensured that most of the causes of variations were considered and the simulations with 10 replications thereof on a population of 2,640 plots, are assumed to give a more nuanced representation of yield responses for the study population and the landscape. However, verification with independent datasets need to be done if the parameters are to be used outside the study region.

In addition to observation to variable ratios, the inclusion of variables in models from a whole set of 64 variables was done by differing specifications. Potential multi-collinearities among continuous variables were screened and only variables that were less correlated were included in the alternative model specifications (see Appendix S4). The Akaike Information Criterion (AIC) and Bayesian Information Criterion (BIC) were used to select the model forms and functions that best modelled farmer SFM decisions and crop productivity [60]. Several other tests were done to ensure validity of the models while capturing variability and at the same time addressing stochasticity. Validity and fitness of the empirical models were determined using measures of goodness of fit. For soil nutrient and SOC spatial distribution, the out of bag error was used [61].

The variability leads to yet another analytical challenge, stochasticity in estimated parameters. Stochasticity existed in the models for estimating nutrient distributions, and for predicting probabilities and intensity of input use, and crop production for the ABM. To ensure that the models are a representation of the real-world phenomena, several combinations of variables and parameters (replications) were used through machine learning algorithms. Uncertainty or stochasticity in soil estimation using the machine learning randomForest model was essentially managed by running tens to hundreds of replications for random subsets based on the knee bend method that indicates stability and validity [62]. The simulation results were also checked for model drift based on differences between the predicted and sampled attribute values. The predictions were adjusted to the baseline real farm conditions using data winzorisation and the model drift coefficients [63].

## Results from first model implementation and discussion

### Impact of fertilizer subsidy on input use and maize yield

The baseline “*current*” scenario has been constructed based on the 2016/2017 subsidy regime averaging 28 % of fertilizer purchased to mimic real-farm conditions and shows predicted fertilizer and manure input and maize yield output (Fig. 6). Given subsidy and existing farm conditions and management, the projected fertilizer usage (kg ha^−1^) increases for the whole population and high input farms (*farmtype3*) but slightly decreases for low and medium input farms (*farmtypes 1& 2*). The manure input is projected to have a stabilising trend while maize yields are projected to decrease. The maize yield gradient is expected as plots that were opened up recently in hillslopes are considered more fertile [64]. But without soil conservation measures [65], productivity is likely to decline if cultivation on these sensitive slopes is continued [66]. Factor elimination revealed that the decreasing trends in maize yields are strongly associated with cultivation period, among other factors.

Given the autonomy that farmers have to decide on nutrient replenishments on their farms [67], they tend to emulate those with similar statuses and aspirations and plan future actions based on their shared experiences. Since the agentic behaviours to anticipate, plan and act are drawn from experiences, their consequences are self-evident. The study revealed that although farmers attribute the behaviours to idiosyncratic abilities, their proactive behaviours are mediated by situational causes such as declining soil fertility where policies such as subsidies may have varying effects.

Juxtaposing the trend graphs, maize is much more responsive to manuring than fertilization. Maize yields for the farms that received both higher fertilizer and higher manure (*farmmtype3*) yielded more maize than those that only received higher fertilizer and moderate manuring (*farmtype2*). This has long-term implications on productivity, nutrient balances and profitability. Yet, manure input is still lower than the levels needed to supply nutrients [68] and far below the requirements to rejuvenate soils [69], while soils under cultivation are on downward degradation spiral [18].

These results are in sharp contrast to the policy expectations. They also depart from existing knowledge established by earlier studies that subsidy induced increases in fertilizer input and economic benefits [[70], [71], [72]]. The recent study by Komarek et al. [72] used the prevailing prices and total nitrogen fertilizer in two scenarios: *full subsidy*, in which case all farmers are assumed to pay zero price, and *zero subsidy* where farmers pay market price. Unlike Komarek's study, the scenarios herein are set based on the assumption that the increases or reductions will be gradual and variable into the foreseeable future, and also contingent on other progressively changing variables being updated as well. Hence, the baseline is set as a trajectory given the prevailing conditions. Worth noting is that during the simulated period, there is a possibility for sudden subsidy regime shifts, which smoothens out over time as has been the case with the previous inducements. This corresponds to the findings by Ricker-Gilber and Jayne [73] who used yearly panel data to estimate temporal effects of subsidy and found that during initial eight years, farmers were conditioned to purchase more fertilizer after three consecutive subsidies. This study, done 15 years after the onset of the current subsidy regime, points to the likelihood that after prolonged exposure to subsidy, some farmers are increasingly attached and become reliant on the program for their fertilizer demand.

Unfortunately, the distribution and acquisition of subsidised fertilizer has been ambiguous. Instead of benefiting the targeted group of the ultra-poor, subsidy has largely benefitted the traders leaving farmers in despair and uncertainty [71]. The ambiguity induced by subsidy has been an antecedent for proactive behaviours by some farmers who, in their pursuit of farming as a major livelihood enterprise, develop agentic capabilities [74].

### Subsidy impacts on balance of soil organic carbon and major nutrients (NPK)

The effects of increasing or decreasing subsidy regimes on nutrient input and output flows have varying implications on nutrient and SOC balance. Given the current subsidy regime and interannual changes in dynamic variables, there is a general increasing trend for NPK balances but a decreasing trend for SOC balances (Fig. 7). The Bonferroni multiple comparison test showed that there are significant differences in the resulting partial N and P balances. The negative impacts on N and P balances from increasing subsidy emanate from the negative effects on fertilization, while the positive shift on K balances among medium input farms is associated with the positive effect on manuring. This underscores the significance of fertilizer as main source of N and P while organic manure is the main source of K [68]. The subsidy policies therefore have potential to impact N and P stocks through fertilization and K stocks through manuring.

The average annual N losses that could be associated with increasing subsidy would be around 7.5 kg ha^−1^ yr^−1^ for medium input farms, which translates to 150 kg ha^−1^ over the simulation period. Increasing the subsidy to universal shifts the average N balance slightly positive with differences from the baseline of 35 and 79 kg ha^−1^ over the simulation period for low input and medium input farms, respectively. The N balances projected for the two polar subsidy regimes favour subsidy removal over universal subsidy with differences of 121, 230 and 259 kg ha^−1^ for low input farms, moderate input farms and the entire population, respectively. Similarly, the P balances are significantly and positively associated with subsidy reduction. On the other hand, universal subsidy leads to a downward shift in P balances for low and medium input farms and for the whole population.

Five decades ago, the soils were naturally productive with 86 % of the farms realising economical yields without fertilizer input because soils had sufficient P and K [[75], [76]]. But as farming was intensified and transitioned from subsistence to market orientation in 1980s, nutrient mining was observed. The government using early results of the trial then, recommended the blanket application of 93 kg N and 40 kg P_2_O_5_ (87 kg ha^−1^ DAP and 175 kg ha^−1^ UREA) [76]. In the 1990s it was noted that at full blanketly recommended rate, 40 % of the farms could not recover the cost of fertilizer [77]. These recommendations are set without considering the supply of NPK from organic manure despite being the major source of K and P in Malawi and N from leguminous plants [[68], [78]].

The current manure and residue inputs of <1 ton ha^−1^ and <5 ton ha^−1^ are below the 8 to 10 ton ha^−1^ annual organic inputs required to bring the soils to levels essential for fertilization response and structural stability [[79], [80]]. These estimations are based on average values, but NetLogo allows identification of individual farms. In this study, some farmers supply 5 t ha^−1^ of manure and could be self-reliant in terms of P and K, and potentially supply the SOC needed for structural stability and long-term productivity of the farms [69].

MASSAI is built as a multi-agent system (MAS) that operationalise the Sustainable Agricultural Intensification (SAI) theoretical framework for spatio-temporal simulation of farm productivity as a coupled human-environmental system. It is built by integrating the core ecological module with human behavioural module to capture, analyse and present nutrient stocks, flows and balances arising from farmer decisions and environmental processes. Considering the major policy intervention, subsidy, and the alternative regimes of universal subsidy or removing it alter the household input budget and behaviour, which impact sustainability outcomes including nutrient input levels, crop productivity and soil nutrient balance. It addresses problems of heterogeneity, interaction, stochasticity, and feedbacks among farming landscapes and land managers.

The study found that within the fragile landscapes of Malawi, farmers strive to utilise the commonly available soil fertility management options as evidenced by wide but low usage. Nine in every ten households used inorganic fertilizers, a third planted legumes and almost half applied manures of various forms. From the empirical and simulated results, it is indicative that the maize mixed smallholder farming system in Malawi has become inelastic to changes in input policies. Despite the changes in subsidy, the total amount of fertilizer applied has levelled off.

The evaluation of proximate and underlying drivers of choice and intensification of inorganic fertilizers, organic manures and legumes has shed light on the diffusion pathways and limitations. Though soil management in smallholder farming systems aims at addressing the most critical nutrient(s), the results from this study show that the soils in some areas are deficient in all three major nutrients (N, P, K) and SOC. Bearing in mind the inter-dependence as expressed by stoichiometry, continuous monitoring and adjustments need to be made to create optimal conditions for plant uptake. Considering the limited capacity on both demand for knowledge of soils by smallholder farmers and the supply of information by agricultural extension services, there is need to raise awareness, capitalise on digital tools to disseminate pixel-based soil information and input these into site-specific nutrient balance and crop yield models.

Subsidy as a major soil nutrient management strategy was viewed by many as a panacea for productivity improvement, a replica of Asia's Green Revolution [81]. The initial impact of subsidy is waning but the fertilizer input levels are still below optimal and the mean yields are far below the optimal or locally attainable yields [82]. Previous studies constructed histories and found, ex post, positive effects of subsidy on maize yields during the initial period [73]. This study highlights the current trends that maize yield has become inelastic to changes among the prevailing interventions. The results suggest that subsidy does not induce farmers to substantially increase fertilization. After 15 years of subsidy, farmers internalised it in their fertilizer expenditure plan, some exclusively relying on subsidy. These input behaviours and productivity trends have implications on nutrient balance and farm sustainability.

## Limitations

This study explored alternative soil management regimes, but the feedback mechanisms to inform policy making are yet to be fully established as indicated below.

1. The parameterisation and the empirical models required for establishing the nutrient stocks and input and output flows are voluminous. Much as efforts were made to extensively review the literature for parameters, and a detailed survey data from representative farms have been used to customize the estimates for the study site, there are still cases where parameters established in other regions and generalised transfer functions have been used. As research in the region progresses, there is need to update the parameters and functions.
2. To evaluate the impact of subsidy, most of the household attributes including labour, women empowerment, household resource endowments as well as plot attributes such as sizes, were not altered or updated. The stochasticity in these variables is captured by random assignment of their coefficient and confidence interval estimates in the SFM choice and production models. Further research should, therefore, build rules for updating these and test their effects by having different scenarios. In the current version of the model, rules for updating labour and gender have been provided and are being incorporated in the model.
3. The modelling environment used, Netlogo, although widely used by ecological and social scientists to model human induced emergent phenomena, its computing capabilities for these small-sized heterogenous farms is limited to extend the model for application to larger landscapes and for other integrated socioecological processes.

## Data Availability

Codes and data are available on request and can be downloaded from https://github.com/powellmponel/MASSAI.

## Appendix Supplementary materials

Image, application 1

## References

[1] Groot J., Oomen G.J.M., Rossing W.A.H. (2012). Multi-objective optimization and design of farming systems. Agric. Syst..

[2] Smaling E.M.A., Fresco L.O. (1993). A decision-support model for monitoring nutrient balances under agricultural land use (NUTMON). Geoderma.

[3] Brundtland Commission (1987).

[4] Pretty J.N. (1997). The sustainable intensification of agriculture. Nat. Resour. Forum.

[5] Laurance W.F., Sloan S., Weng L., Sayer J.A. (2015). Estimating the environmental costs of Africa's Massive “development Corridors. Curr. Biol..

[6] Rockström J., Steffen W., Noone K., Persson Å., Chapin F.S., Lambin E.F., Lenton T.M., Scheffer M., Folke C., Schellnhuber H.J., Nykvist B., De Wit C.A., Hughes T., Van Der Leeuw S., Rodhe H., Sörlin S., Snyder P.K., Costanza R., Svedin U., Falkenmark M., Karlberg L., Corell R.W., Fabry V.J., Hansen J., Walker B., Liverman D., Richardson K., Crutzen P., Foley J.A. (2009). A safe operating space for humanity. Nature.

[7] Giller K.E., Beare M.H., Lavelle P., Izac A.-M.N., Swift M.J. (1997). Agricultural intensification, soil biodiversity and agroecosystem function. Appl. Soil Ecol..

[8] Scoones I. (1998).

[9] Smith A., Snapp S., Chikowo R., Thorne P., Bekunda M., Glover J. (2017). Measuring sustainable intensification in smallholder agroecosystems : a review. Glob. Food Secur..

[10] Weltin M., Zasada I., Piorr A., Debolini M., Geniaux G., Moreno Perez O., Scherer L., Tudela Marco L., Schulp C.J.E. (2018). Conceptualising fields of action for sustainable intensification – A systematic literature review and application to regional case studies. Agric. Ecosyst. Environ..

[11] Giller K.E., Tittonell P., Rufino M.C., van Wijk M.T., Zingore S., Mapfumo P., Adjei-Nsiah S., Herrero M., Chikowo R., Corbeels M., Rowe E.C., Baijukya F., Mwijage A., Smith J., Yeboah E., van der Burg W.J., Sanogo O.M., Misiko M., de Ridder N., Karanja S., Kaizzi C., K'ungu J., Mwale M., Nwaga D., Pacini C., Vanlauwe B. (2011). Communicating complexity: Integrated assessment of trade-offs concerning soil fertility management within African farming systems to support innovation and development. Agric. Syst..

[12] The Montpellier Panel (2013).

[13] Matson P.A., Matson A P., Parton J W., Power G A., Swift J M. (1997). Agricultural intensification and ecosystem properties. Science.

[14] Schreinemachers P., Berger T. (2011). An agent-based simulation model of human–environment interactions in agricultural systems. Environ. Model. Softw..

[15] Boulanger P.M., Bréchet T. (2005). Models for policy-making in sustainable development: The state of the art and perspectives for research. Ecol. Econ..

[16] Sanchez P.A. (2002). Soil fertility and hunger in Africa. Science.

[17] Vanlauwe B., Six J., Sanginga N., Adesina A.A. (2015). Soil fertility decline at the base of rural poverty in sub-Saharan Africa. Nat. Plants.

[18] Mpeketula P.M.G. (2016).

[19] Berger T. (2001). Agent-based spatial models applied to agriculture: a simulation tool for technology diffusion, resource use changes and policy analysis. Agric. Econ..

[20] Le Q.B., Park S.J., Vlek P.L.G.G., Cremers A.B. (2008). Land-use dynamic simulator (LUDAS): a multi-agent system model for simulating spatio-temporal dynamics of coupled human–landscape system. I. Structure and theoretical specification. Ecol. Inform..

[21] Miyasaka T., Le Q.B., Okuro T., Zhao X., Takeuchi K. (2017). Agent-based modeling of complex social–ecological feedback loops to assess multi-dimensional trade-offs in dryland ecosystem services. Landsc. Ecol..

[22] Schindler J., Vlek L, G P., Ehlers E (2009).

[23] Villamor G.B., Le Q.B., Djanibekov U., van Noordwijk M., Vlek P.L.G. (2014). Biodiversity in rubber agroforests, carbon emissions, and rural livelihoods: An agent-based model of land-use dynamics in lowland Sumatra. Environ. Model. Softw..

[24] Railsback S.F., Grimm V. (2012).

[25] Ferber J. (1999).

[26] J. Groot, G. Oomen, Farm DESIGN Manual - Version 4.21.0, 2018, Farming Systems Ecology Group, Wageningen University and Research, The Netherlands, (At: https://www.scribd.com/document/379689984/Farm-DESIGN-Manual Accessed: 25/02/2019)

[27] Tamene L., Adimassu Z., Aynekulu E., Yaekob T. (2017). Estimating landscape susceptibility to soil erosion using a GIS-based approach in Northern Ethiopia. Int. Soil Water Conserv. Res..

[28] H.A. Bootsma, J. Mwita, B. Mwichande, R.E. Hecky, J. Kihedu, J. Mwambungu, The Atmospheric Deposition of Nutrients on Lake Malawi/Nyasa, In: H.A. Bootsma, R.E. Hecky, (Eds), Water Quality Report, Lake Malawi/Nyasa Biodiversity Conservation Project, DSADC/GEF, 1999, pp. 85-111, (At: https://bpb-us-w2.wpmucdn.com/sites.uwm.edu/dist/e/211/files/2020/11/Ch3-Atmospheric-Nutrient-Deposition.pdf).

[29] Lesschen J.P., Stoorvogel J.J., Smaling E.M.A.A., Heuvelink G.B.M.M., Veldkamp A. (2007). A spatially explicit methodology to quantify soil nutrient balances and their uncertainties at the national level. Nutr. Cycling Agroecosyst..

[30] Mloza-Banda M.L., Cornelis W.M., Mloza-Banda H.R., Makwiza C.N., Verbist K. (2014). Soil properties after change to conservation agriculture from ridge tillage in sandy clay loams of mid-altitude Central Malawi. Soil Use Manag..

[31] Ibrahim A., Pasternak D., Fatondji D. (2015). Impact of depth of placement of mineral fertilizer micro-dosing on growth, yield and partial nutrient balance in pearl millet cropping system in the Sahel. J. Agric. Sci..

[32] Scholz W.R., Binder R.C., Lang J.D. (2011). Environmental Literacy in Science and Society: From Knowledge to Decisions.

[33] Quang D.V., Schreinemachers P., Berger T. (2014). Ex-ante assessment of soil conservation methods in the uplands of Vietnam: An agent-based modeling approach. Agric. Syst..

[34] Mponela P., Villamor G.B., Snapp S., Tamene L.D., Lee Q.B., Borgemeister C. (2020). Digital soil mapping of nitrogen, phosphorus, potassium, organic carbon and their crop response thresholds in smallholder managed escarpments of Malawi. Appl. Geogr..

[35] Mponela P., Villamor G.B., Snapp S., Tamene L.D., Lee Q.B., Borgemeister C. (2020). The role of women empowerment and labour dependency on adoption of integrated soil fertility management in Malawi. Soil Use Manag..

[36] Tittonell P., Muriuki A., Shepherd K.D., Mugendi D., Kaizzi K.C., Okeyo J., Verchot L., Coe R., Vanlauwe B. (2010). The diversity of rural livelihoods and their influence on soil fertility in agricultural systems of East Africa – A typology of smallholder farms. Agric. Syst..

[37] Berger T., Schreinemachers P. (2006). Creating Agents and Landscapes for Multiagent Systems from Random Samples. Ecol. Soc..

[38] Lowole W.M. (1965). Soil map of Malawi. National Soil Maps.

[39] FAO (2012).

[40] U.S. Geological Survey, contains modified Copernicus Sentinel 2 data, processed by ESA, 2018a (At: https://earthexplorer.usgs.gov/. Accessed: 20/5/2018).

[41] U.S. Geological Survey, SRTM DEM data, 2018b (At: https://earthexplorer.usgs.gov/. Accessed: 20/5/2018).

[42] Tittonell P., Giller K.E. (2013). When yield gaps are poverty traps: the paradigm of ecological intensification in African smallholder agriculture. Field Crops Res..

[43] NEPAD (2006).

[44] Chirwa E.W., Dorward A., Matita M. (2011). Initial conditions and changes in commercial fertilizers under the farm input subsidy programme in Malawi: implications for graduation. Future Agric..

[45] Dorward A., Chirwa E. (2011). The Malawi agricultural input subsidy programme: 2005-6 to 2008-9. Int. J. Agric. Sustain. IJAS.

[46] GoM, National fertiliser strategy, Ministry of Agricultre, Lilongwe, Malawi, 2007 (At: http://fsg.afre.msu.edu/mgt/caadp/format_for_national_fertilizer_strategy9.pdf. Accessed: 23/10/2018).

[47] IFDC (2013).

[48] Ricker-Gilbert J., Mason N.N., Jayne T.S., Darko F., Tembo S. (2013).

[49] Damania R., Balseca E., de Fontaubert C., Gill J., Kim K., Rentschler J., Russ J., Zaveri E. (2023).

[50] Holden S., Lunduka R. (2012). Do fertilizer subsidies crowd out organic manures? The case of Malawi. Agric. Econ..

[51] Wilensky U. (1999). NetLogo.

[52] Greenland S., Robins J.M., Pearl J. (1999). Confounding and collapsibility in causal inference. Stat. Sci..

[53] Wooldridge J.M. (2012).

[54] G.B. Villamor, Flexibility of multi-agent system models for rubber agroforest landscapes and social response to emerging reward mechanism for ecosystems services in Sumatra, Indonesia, PhD Thesis, Rheinischen Friedrich-Wilhelms-Universität Bonn, 2012 (At: https://hss.ulb.uni-bonn.de/2012/2865/2865.pdf).

[55] Bandura A. (1986).

[56] Greenland S., Pearl J., Robins J.M. (1999). Causal diagrams for epodemioloc research. Epidemiology.

[57] Thiele J.C., Kurth W., Grimm V., Thiele Jan C., Kurth Winfried, Grimm Volker (2014). Facilitating parameter estimation and sensitivity analysis of agent-based models : a cookbook using NetLogo and R. J. Artif. Soc. Soc. Simul..

[58] Hochberg Y. (1988). A sharper bonferroni procedure for multiple tests of significance. Biometrika.

[59] Austin P.C., Steyerberg E.W. (2015). The number of subjects per variable required in linear regression analyses. J. Clin. Epidemiol..

[60] Vrieze S.I. (2012). Model selection and psychological theory: a discussion of the differences between the Akaike information criterion (AIC) and the Bayesian information criterion (BIC). Psychol. Methods.

[61] Breiman L. (2001). Random forests. Mach. Learn..

[62] A. Liaw, M. Wiener, Breiman and cutler's random forests for classification and regression, R-Package, 2018, (At: https://cran.r-project.org/web/packages/randomForest/randomForest.pdf. Accessed: 20/02/2019).

[63] Donkin E., Dennis P., Ustalakov A., Warren J., Clare A. (2017). Replicating complex agent based models, a formidable task. Environ. Model. Softw..

[64] Braslow J., Cordingley J. (2016). Participatory mapping in Ntcheu district, Malawi. A case study. Int. Cent. Trop. Agric. CIAT.

[65] CIAT (2016).

[66] Banda A.Z., Maghembe J.A., Ngugi D.N., Chome V.A., Banda Z A., Maghembe A J., Ngugi N D., Chome A V. (1994). Effect of intercropping maize and closely spaced Leucaena hedgerows on soil conservation and maize yield on a steep slope at Ntcheu, Malawi. Agrofor. Syst..

[67] Sambo P.T., Haywood C., Wardell D.A., Kibugi R., Cordonnier-Segger M.C. (2015).

[68] Chilimba A.D.K., Shano B., Chigowo M.T., Komwa M.K. (2005). Quality assessment of compost manure produced by smallholder farmers in Malawi.

[69] Zingore S., Tittonell P., Corbeels M., van Wijk M.T., Giller K.E. (2011). Managing soil fertility diversity to enhance resource use efficiencies in smallholder farming systems: a case from Murewa District, Zimbabwe. Nutr. Cycling Agroecosyst..

[70] Arndt C., Pauw K., Thurlow J. (2016). The economy-wide impacts and risks of Malawi's farm input subsidy program. Am. J. Agric. Econ..

[71] Holden S.T., Lunduka R.W. (2013). Who benefit from Malawi's targeted farm input subsidy program?. Forum Dev. Stud..

[72] Komarek A.M., Drogue S., Chenoune R., Hawkins J., Msangi S., Belhouchette H., Flichman G. (2017). Agricultural household effects of fertilizer price changes for smallholder farmers in central Malawi. Agric. Syst..

[73] Ricker-Gilbert J., Jayne T.S. (2017). Estimating the enduring effects of fertiliser subsidies on commercial fertiliser demand and maize production: panel data evidence from Malawi. J. Agric. Econ..

[74] Grant A.M., Ashford S.J. (2008). The dynamics of proactivity at work. Res. Organ. Behav..

[75] Mutegi J., Kabambe V., Zingore S., Harawa R., Wairegi L. (2015).

[76] Snapp S. (1998). Soil nutrient status of smallholder farms in Malawi. Commun. Soil Sci. Plant Anal..

[77] Benson T.D., Ransom J.K., Palmer A.F.E., Zambezi B.T., Mduruma Z.O., Waddington S.R., Pixley K.V., Jewell D.C. (1996).

[78] Palm C., Giller K.E., Mafongoya L.P., Swift J.M. (2001). Management of organic matter in the tropics: translating theory into practice. Nutr. Cycling Agroecosyst..

[79] Musinguzi P., Ebanyat P., Tenywa J.S., Basamba T.A., Tenywa M.M., Mubiru D.N. (2016). Critical soil organic carbon range for optimal crop response to mineral fertiliser nitrogen on a ferralsol. Exp. Agric..

[80] Musinguzi P., Tenywa J.S., Ebanyat P., Tenywa M.M., Mubiru D.N., Basamba T.A., Leip A., Patrick M., Tenywa J.S., Ebanyat P., Tenywa M.M., Mubiru D.N., Basamba T.A., Leip A. (2013). Soil organic carbon thresholds and nitrogen management in tropical agroecosystems: concepts and prospects. J. Sustain. Dev..

[81] Denning G., Kabambe P., Sanchez P., Malik A., Flor R., Harawa R., Nkhoma P., Zamba C., Banda C., Magombo C., Keating M., Wangila J., Sachs J. (2009). Input subsidies to improve smallholder maize productivity in Malawi: toward an African Green Revolution. PLoS Biol..

[82] Tamene L.D., Mponela P., Ndengu G., Kihara J. (2016). Assessment of maize yield gap and major determinant factors between smallholder farmers in the Dedza district of Malawi. Nutr. Cycling Agroecosyst..

