# Supplementary material for: MASSAI: Multi-agent system for simulating sustainable agricultural intensification of smallholder farms in Africa
Source: MethodsX. 2023 Oct 30;11:102467. doi: 10.1016/j.mex.2023.102467 (PMC10643297; doi:10.1016/j.mex.2023.102467)
Supplement: Supplementary file 1 [file mmc1.docx]

# Supplementary

Table S1 The ODD+D documentation of the MAS for simulating SAI in rural Malawi

|  | **Contents** | **Description** |
| --- | --- | --- |
| **Overview** | | |
| Purpose | Question, problem or hypothesis (i.e. overall objective) | The purpose of the MASSAI is to understand how usage of chemical and biological soil management interventions under government subsidy impacts short-term yields and long-term nutrient balances and the resulting structural changes in farming systems and household incomes. |
| Entities, state variables and scales | Kind of entities, their attributes (include units), spatial and temporal resolutions | Human agent: farmers with heterogeneous demographic characteristics (age, number of members, labour), economic activities (income from farm and non-farm), land ownership and the parameters for soil fertility technology choice probabilities and usage intensities.  Soil agent: the top soil (0-30cm) actively explored by annual crops for nutrients and water. Its characteristics includes soil properties (NPK and SOC), which though transfer functions that capture nutrient dynamics responds to natural and human influence.  Plant agent: refers to crops grown on a farm parcel and is characterised by parcel location, area, crop type, farming practices, yield and nutrient contents. It responds to dynamics in soil and human actions.  These three agents make up the ‘farm’ with linked agronomic, ecological and economic performance indicators. |
|  |  | Spatial units: georeferenced raster with grid cells 10m x 10m and the heterogeneous environmental assets and drivers: natural (soil properties, topography, vegetation, and the co-efficient of variables for nutrient transfer dynamics sub-models), agricultural (land-use – cultivated or not use, area, agricultural yield, nutrient inputs, labour force, parameters for agricultural yield and nutrient transfer dynamics sub-models), and institutional (ownership, village) |
| Process overview and scheduling | What entities does and in what order? | Soil nutrient input strategy choice and use intensity:  Nutrient stockpiles and transfer modules: Biophysical driven processes of nutrient supply, transformation, transport and export mediated by ecological processes and human activity.  Land use choice: Crop choice probabilities (sole cereal or cereal plus legume); Rule based determination of land parcels switching from agriculture to woodland/grassland and vice versa. |
| **Design concept** | | |
| Basic principles | Which concepts, theories and hypotheses underlie the model design? | Theoretically, we adopt the farm styles theory which purports that the farm and landscape structural changes are spatio-temporal explicit dynamic processes emerging from individual farmers decisions and actions about farm inputs and their perceptions and control of farm outputs on each of the land parcels (van der Ploeg & Long, 1990). To explicitly capture and formalise the linkages and feedbacks between ecological processes and human actions, we adapt the Human-Environmental Framework (Scholz *et al.*, 2011) as indicated in Figure 2.1. We use the indicators and the metrix of sustainable intensification for smallholder farmers (Smith *et al.*, 2015), and the nutrient input and output transfer functions as formulated in farmDesign (Groot *et al.*, 2012) and nutrient monitoring farm models (Smaling & Fresco, 1993).  The farmer decision model is based on micro-economic theory, with the assumption that farmers are private entities and use the soil technologies to maximize utility from land units. To do so they either enhance productivity or reduce degradation hence the decisions are: (i) rational by maximizing yields; (ii) bounded rationality of input use according to average benefits; or (iii) ad-hoc rules such as applying due to availability and/or access to fertilizer. In some cases, there are abrupt system changes such as burning crop residues, which were previously incorporated, to control fall armyworms. The double hurdle model is chosen because the aim is to analyse the factors influencing household’s probability and extent of soil fertility management. The Sustainable Livelihood Framework (SLF) was used to take stock of factors that influence farmers’ decisions and abilities to undertake practices for a particular livelihood strategy (Scoones, 1998). Data was captured through soil, crop yield, household surveys that were geo-linked using a common sample frame.  The landscape dynamics and processes are formulated based on the principle of ecological equilibrium. As populated by (Stoorvogel & Smaling, 1990), there are five inflows and outflows of inputs and outputs. Of the five ins and outs, 3 are archetypal ecological, and in pristine ecosystem we envisage that nutrient flows and stocks are in ecological equilibrium. However, for managed agricultural systems, two of the inputs and two outputs are largely while others partially mediated by human action and the system’s equilibrium shifts after a series of disturbances. |
| Emergence | What model results are expected to vary when characteristics of individuals and environment change? Vis -a-vis results imposed by model rules (build in). | Framed by constraints and opportunities, actions by individual smallholder farmers when aggregated over space and accumulated over time become an unformidable force that continually shapes the environment-community agricultural productivity. The recurrent low crop yield, hunger, poverty, low input use, low yield cycle is a typical phenomenon in most parts of Malawi, creating vicious cycles of poverty traps (Tittonell & Giller, 2013). Aggregation and shifts are expected in land use expressed as crop(s) planted, nutrient input and output, soil nutrient stocks, crop yields, farm incomes and number of farmers adopting nutrient input strategies. |
| Adaptation | Do the individuals make decisions/ behavior to achieve objectives e.g. change cells | The agents change parameters or objective function defining the behaviour after acquisition of experience during the model lifespan (in this case 1 year). The parameters for the household’s resource allocation for soil fertility improvement may change depending on farm performance. The farming household may transform (move to another farm type) when their objective function, thus the resource allocation rules as well as the parameters, changes.  For continuous cropping, the plant production function remains the same but parameters changes. In rotation, both parameters and production functions changes overtime. |
| Objectives | Individuals success is a result of adaptive traits. Criteria used for ranking alternatives. | Agents exploit, control and consume entities and resources from their own plots and those of the surrounding environment to achieve organisational goal. Each farming household within the community is assumed to have specific state variables that enable them to make autonomous decisions regarding the improvement of soil fertility of their plots at a particular time.  Proactively and opportunistically, take actions to achieve its goals given the dynamic and unpredictable environment. The households make decisions depending on the current and expected soil fertility of their farm bounded by the resources available with the aim of staying focused on achieving own objectives. Given many SLM options, the farmers allocate resources to either the one that maximises soil fertility improvement or the one that minimises risk of soil degradation. |
| Learning | Collective or individual experience resulting in change in adaptive traits. | The demonstration plots are usually set along the roads hence we assume that most farmers learn from those with plots along the community paths. |
| Prediction | Models for future conditions or consequences that individuals use for successful decision making | Perceptive: considered to have true scientific knowledge (or if information is limited, belief) of the environment.  The household’s perception (vision) of soil fertility though referenced by others in community, efforts to improve it are restricted by tenure to own plots and not the entire landscape. However, for landscape processes such as soil erosion on hill slopes, they also mainly recognise activities upslope and in rare cases downslope in case of an extending gully. |
| Sensing | Internal, neighbours, and environmental state variables that individuals sense and consider in their behaviour. Local, networks or global levels. Information acquisition.  Accuracy and uncertainity. | Households exploits the farms for ecosystem services that are dependent on soil status, radiation, rainfall and temperature. Humans controls the land through among many things, managing soil fertility and controlling soil erosion. Their search area is constrained by (im)mobility and, for the established settlements, by use rights.  Thus, the possibility of accessing and manipulating the existing environmental entities and resources is often bounded. Sensing is constrained within the agent class and also local neighbourhood (village boundary).  The actions of the agents on real farms are non-deterministic with some degree of uncertainty. The main processes for soil fertility improvement and erosion control will be used to represent the environment. The choice of SLM technologies will be depended on their knowledge about availability and performance of the technologies in the study area (Zambonelli *et al.*, 2003). |
| Interaction | Direct or indirect (e.g competition for mediating resource). Do they communicate? | Interactive: achieve goals by interacting with other agents in the environment the agent is situated.  Individuals do not conform to set land management rules, but since some objectives of SLM are realised at larger community and landscape level, farmers co-operate with others through social ties or shared landscapes.  Given their socioeconomic capabilities and land potential farmers tend to mimic those with similar typology (Le, 2005).  The interactions are bounded by village boundary and shared landscapes. |
| Heterogeneity | Do the agents and landscapes differ | The study population is comprised of farmers with different demographic (sex, age), endowments (land, labour) and location (dwelling and plot ownership). The environmental attributes that are variable among pixels (plots) include topography, base soil fertility, and crop biomass/yield. |
| Stochasticity | Processes assumed to be random. Used to reproduce variability for processes difficult to capture actual. | The remaining population and their plots are randomly allocated to non-sampled cultivated grids. Their attributes are drawn from the sampled households and plots to generate a population which mimics the distribution of statistics found in real farms.  Some of the environmental attributes such as terrain are set to be static i.e. remain unchanged without the action of the agents. However, most of the entities and resources are active and dynamic with changes that are beyond the control/regulation of the individual agents. Geo-simulation of dynamic soil fertility is one required end but quite variable hence is randomly set in most cases using the estimated confidence limits. |
| Collectiveness | Belonging or forming groups: defined by modeler or result of individual behaviours. | The individual farmers actions influence others in the system although not explicitly modelled, but through social ties and shared landscapes, farmers tend to learn from and imitate those with the same typology.  Households often apply soil fertility improvement technologies on individual plots as discrete entities. However, for technologies that aim at controlling soil erosion such as permanent vegetation cover, the environment needs to be viewed as a continuous entity with connected discrete entities.  The households belonging to a typology have similar resource endowments that they use to pursue similar livelihood strategies (bounded by policies and institutions). The typologies are thus used to initialise the agent population and households transition depending on resource accumulation or depletion by end of the activity calender. |
| Observation | Data collected from ABM | The simulated outputs, which is the projected development, are compared with the baseline and other past states in terms of changes in structure (e.g. distribution of farm types) and function (e.g. nutrient balance) of the farms. |
| **Details** | | |
| Implementation | How has the model been implemented | Using the multi agent platform Netlogo (Wilensky, 1999), the first step has been to adapt the LUDAS modules (Le, 2005) for initialization, decision, agricultural production, and crop allocation. Empirical models and transfer functions have been used to frame local processes and estimate parameters for the study site. |
| Initialization | Initial state of the model world at time 0 of a simulation run (exact or stochastically set). | Baseline soil nutrient levels, plot sizes, distribution and productivity, and farmer attributes, nutrient stocks, input and outputs (exact for the sample and randomly allocated for the rest). Ecological inputs and outputs estimated using transfer functions.  For subsequent runs initial conditions are the same among simulations, the stochastic attributes are estimated using the random number generator with a certain confidence interval and random seed. |
| Input data | External data files or models to represent processes | The external data and models are used to set initial state and for parameterization of processes. These include satellite imagery and parameters for the transfer functions. |
| Sub-models | Detailed process overview and scheduling | Sub-models are presented in respective the methodology sections. The empirical results and estimations are systematically implemented in NetLogo using the main directory (subdirectory) structure. The initialisation and static processes are one-step time whilst the dynamic processes are set to run for a one-year cycle corresponding to the unimodal production season. |
